# Prevalence, characterization, and antibiotic susceptibility of *Vibrio parahaemolyticus* isolated from retail aquatic products in North China

**DOI:** 10.1186/s12866-016-0650-6

**Published:** 2016-03-09

**Authors:** Xiaoke Xu, Jianheng Cheng, Qingping Wu, Jumei Zhang, Tengfei Xie

**Affiliations:** State Key Laboratory of Applied Microbiology Southern China, Guangdong Provincial Key Laboratory of Microbial Culture Collection and Application, Guangdong Institute of Microbiology, No. 100 Central Xianlie Road, Guangzhou, 510070 China

**Keywords:** Antimicrobial sensitivity, Aquatic products, ERIC-PCR, Prevalence, Quantitative analysis, Serotype, *Vibrio parahaemolyticus*, Virulence gene

## Abstract

**Background:**

*Vibrio parahaemolyticus* is a major foodborne pathogen, particularly in Asian countries. Increased occurrence of outbreaks of *V. parahaemolyticus* gastroenteritis in China indicates the need to evaluation of the prevalence of this pathogenic species. *V. parahaemolyticus* distribution in shellfish from the eastern coast of China has been reported previously. However, to date, the prevalence of *V. parahaemolyticus* in retail aquatic products in North China has not been determined. To investigate the prevalence of *V. parahaemolyticus* in aquatic products in North China, 260 aquatic product samples were obtained from retail markets in 6 provinces of North China from November to December in 2012 and July to August in 2013.

**Results:**

*V. parahaemolyticus* was detected in 94 (36.2 %) of the samples by the most probable number method. The density of *V. parahaemolyticus* ranged from 1.50 to 1100 MPN/g. *V. parahaemolyticus* was detected at a rate of 50.0 % and 22.7 % in summer and in winter, respectively. The density of *V. parahaemolyticus* was significantly higher in summer than in winter, with mean levels of 16.5 MPN/g and 5.0 MPN/g, respectively. Among 145 *V. parahaemolyticus* isolates examined, none of the isolates possessed *tdh* and *trh*. In multiplex PCR-based O-antigen serotyping of these 145 isolates, all serotypes, other than O6, O7, and O9, were detected, and serotype O2 was found to be the most prevalent (detected in 54 isolates). The 145 isolates were grouped into 7 clusters by enterobacterial repetitive intergenic consensus-polymerase chain reaction (ERIC-PCR) at a similarity coefficient of 0.66. The antimicrobial resistance patterns of these 145 isolates to 12 antimicrobial agents revealed that most of the isolates were resistant to streptomycin (86.2 %), while fewer were resistant to ampicillin (49.6 %), cefazolin (43.5 %), cephalothin (35.9 %), and kanamycin (22.1 %). All of the examined isolates were susceptible to azithromycin and chloramphenicol.

**Conclusions:**

The findings of this study will help in defining appropriate monitoring programs, understanding of the dissemination of antibiotic resistant strains, and providing information for the assessment of exposure to this microorganism at the consumption level.

**Electronic supplementary material:**

The online version of this article (doi:10.1186/s12866-016-0650-6) contains supplementary material, which is available to authorized users.

## Background

*Vibrio parahaemolyticus* is a human pathogen that has been associated with gastroenteritis worldwide [1–4], and outbreaks have been reported in many countries such as the USA, France, and New Zealand [5–7]. Moreover, in recent years, *V. parahaemolyticus* has been reported as a significant cause of foodborne bacterial poisoning in China [8, 9].

*V. parahaemolyticus* has been isolated from samples of a variety of aquatic products, including fish, shrimp, oyster, and clam [10, 11], and it is among the most common causative agents of aquatic product-associated gastroenteritis in the world [3, 12–16]. With the vigorous development of the Chinese economy, there has been a rapid increase in aquatic product consumption, not only along the coast of China, but also in mainland China. *V. parahaemolyticus* distribution in shellfish from the eastern coast of China has been reported previously [17]. Our previous studies have also shown that shrimp in Chinese retail markets are contaminated with *V. parahaemolyticus* [18]*.* However, to date, the presence of *V. parahaemolyticus* in retail aquatic products in North China has received less attention, and little information is available on the prevalence and contamination levels of *V. parahaemolyticus* in such aquatic products. Although *V. parahaemolyticus* is frequently present in aquatic products, most strains of this species are nonpathogenic to humans [19]; however, virulent *V. parahaemolyticus* strains are clearly a concern for aquatic product safety.

Detection of pathogenic *V. parahaemolyticus* isolates is typically based on molecular biological analysis that amplify *tdh* and *trh* sequences [20, 21]. These 2 genes, encoding the thermostable direct hemolysin (TDH) and the homologous thermostable direct hemolysin-related hemolysin (TRH), respectively, have been implicated in *V. parahaemolyticus* virulence [22–26]. However, a recent study showed that pathogenesis of *V. parahaemolyticus* does not appear to rely solely on a given virulence function; rather, virulence is a complex trait and different strains may employ somewhat different strategies [1].

To date, on the basis of somatic (O) and capsular (K) antigens, *V. parahaemolyticus* is classified into 13 O-serogroups and 71 K-serogroups [27, 28]. Serotyping has been widely used for identifying isolates in epidemiological studies. Furthermore, certain *V. parahaemolyticus* serotypes have been considered to be more virulent than others [29, 30]. A multiplex PCR-based O-antigen serotyping method for *V. parahaemolyticus* has been successfully developed [31]. Therefore, PCR-based serotyping is considered a convenient method for the rapid and accurate identification of a wide array of *V. parahaemolyticus* isolates. However, serotyping offers limited information about the genetic relatedness of strains.

In addition to serotyping, a variety of molecular typing methods have been applied to characterization of *V. parahaemolyticus*. Molecular typing of *V. parahaemolyticus* was shown to be a useful tool for providing information about the genetic relatedness of strains and for detection of virulent strains [32]. In recent years, a number of typing methods such as pulsed-field gel electrophoresis (PFGE) [33], ribotyping [34], random amplified polymorphic DNA (RAPD) analysis [35, 36], multi-locus sequence typing (MLST) [37], and enterobacterial repetitive intergenic consensus-polymerase chain reaction (ERIC-PCR) [38, 39] have been applied in the typing of *V. parahaemolyticus*. ERIC-PCR has previously proven useful for subtyping *V. parahaemolyticus* [33, 38, 39], and has been successfully used for genotyping different bacterial pathogens in previous studies [40–42].

Antimicrobials are commonly used in the treatment of infectious diseases in the aquaculture industry; however, the extensive use of antimicrobials has led to the development of antimicrobial resistance among pathogens in aquatic products and has rendered many known antimicrobials ineffective. *V. parahaemolyticus* has been reported to have resistance to ampicillin, streptomycin, kanamycin, tetracycline, and ciprofloxacin [43–46]. Antimicrobial resistance, particularly multi-drug resistance, is among the most important public health concerns because it is directly related to disease management and control [47, 48]. Therefore, it is necessary to establish a monitoring system for the objective evaluation of the antimicrobial-resistance profile.

Therefore, the objective of this study was to investigate the seasonal prevalence and levels of *V. parahaemolyticus* in retail aquatic products in North China. The virulence, serological types, and ERIC types were focused on, and the antibiotic resistance patterns of the isolated strains were determined.

## Results

### *V. parahaemolyticus* in aquatic products

The prevalence of *V. parahaemolyticus* in the 260 aquatic product samples examined in this study is shown in Table 1. *V. parahaemolyticus* was detected in 94 (36.2 %) of the 260 samples. Among the positive samples, the prevalence of *V. parahaemolyticus* were 23.4 % (22/94) in fish samples and 43.4 % (72/166) in shrimp samples. The density of *V. parahaemolyticus* varied from 1.50 to 1100 MPN/g. The mean levels of the pathogen in fish and shrimp samples were 14.0 MPN/g and 8.7 MPN/g, respectively. Independent-samples *t*-test analysis of *V. parahaemolyticus* levels versus 2 kinds of aquatic product samples indicated no statistically significant differences (*P* = 0.190).

**Table 1:** Prevalence and levels of *Vibrio parahaemolyticus* in retail aquatic products from North China

Aquatic products samples	No. of samples analyzed	No. of samples positive (%)	No. of samples containing the pathogen (MPN/g)			
			3 to 10	>10 to 10^2^	>10^2^ to 10^3^	>10^3^
Fish	94	22 (23.4)	18	3	0	1
Shrimp	166	72 (43.4)	50	19	3	0
Total	260	94 (36.2)	68	22	3	1

In seasonal distribution, the maximum isolation rate of *V. parahaemolyticus* in aquatic products was in summer, and reached 50.0 %, while it was 22.7 % in winter (Table 2). The mean levels of *V. parahaemolyticus* in samples collected during summer and winter were 16.5 MPN/g and 5.0 MPN/g, respectively, which was significantly different (*P* = 0.040).

**Table 2:** Prevalence and levels of *Vibrio parahaemolyticus* in retail aquatic products from North China during different seasons

Season	No. of samples analyzed	No. of samples positive (%)	No. of samples containing the pathogen (MPN/g)			
			3 to 10	>10 to 10^2^	>10^2^ to 10^3^	>10^3^
Winter	132	30 (22.7)	25	4	1	0
Spring	128	64 (50.0)	43	18	2	1
Total	260	94 (36.2)	68	22	3	1

### Detection of *tdh* and *trh* genes in *V. parahaemolyticus* isolates

In total, 145 *V. parahaemolyticus* isolates were confirmed and tested for the presence of *trh* and *tdh*. None of the isolates possessed these genes.

### O-serogroup typing by multiplex PCR

With the exception of serotypes O6, O7, and O9, all other serotypes were detected among the isolates. Serotype O2 was the most prevalent (54 isolates), followed by serotype O1 (25 isolates). The results of the O-antigen serotyping for all 145 isolates are shown in Table 3 and Additional file 4: Table S1.

**Table 3:** Results of the PCR-based O-antigen serotyping of 145 *Vibrio parahaemolyticus* isolates

Serogroups		Product sizes (bp)	No. of isolates analyzed
Group 1	O1	474	25
	O2	238	54
	O4	671	5
	O5	852	6
	O10	343	2
Group 2	O3^a^	868	8
	O8	680	11
	O11	524	17
	O12	256	6
	Uncertain		11
Total			145

^a^O3 or O13

### ERIC-PCR

**Fig 1:**
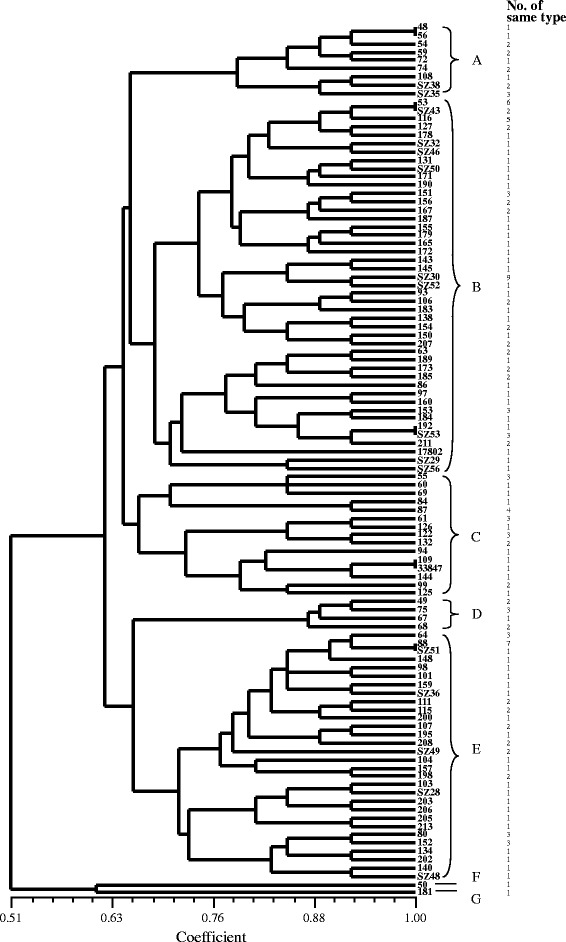
ERIC-PCR DNA fingerprint analysis of *Vibrio parahaemolyticus* isolates in retail aquatic products from North China

The results of ERIC-PCR analysis of the 145 isolates are shown in Fig. 1. ERIC-PCR resulted in 4 – 10 amplification bands, with a size ranging from 130 bp to about 6000 bp. Bands with molecular sizes of 500, 1500, and 2500 bp were common to most isolates (Additional file 1: Figure S1, Additional file 2: Figure S2, and Additional file 3: Figure S3). Only 1 strain was represented in the figure if more than 2 strains of the same isolate type were analyzed. At a relative similarity coefficient of 0.66, the 145 isolates were classified into 7 clusters (designated as A, B, C, D, E, F, and G). Most isolates were distributed between the B and E clusters. One isolate (NO. 109) and a reference strain (ATCC 33847) were grouped into the same cluster; and some isolates (NOs. 53, 88, and 192) and clinical strains (NOs. SZ43, SZ53, and SZ51) were grouped into the same cluster, respectively.

### Antimicrobial susceptibility

Isolates of *V. parahaemolyticus* were tested for different levels of antibiotic resistance. The isolates were most resistant to streptomycin, with resistance and intermediate rates of 86.2 % and 11.7 %, respectively. In addition, the isolates exhibited relatively high resistance rates, of 49.6 %, 43.5 %, 35.9 %, and 22.1 %, for ampicillin, cefazolin, cephalothin, and kanamycin, respectively. However, it was surprising to note that strain 58, isolated from a fish sample, was a multi-drug-resistant strain, which showed resistance to 7 antibiotics: streptomycin, cephalothin, ampicillin, tetracycline, kanamycin, trimethoprim-sulfamethoxazole, and cefazolin. All of the examined isolates were susceptible to azithromycin and chloramphenicol. Among the remaining tested antibiotics, the next-highest susceptibility rates were observed for nalidixic acid (97.2 %), ciprofloxacin (91.7 %), tetracycline (83.4 %), trimethoprim-sulfamethoxazole (75.2 %), and gentamicin (62.8 %). The susceptibility, intermediate resistance, and resistance rates of the 145 examined *V. parahaemolyticus* isolates with respect to 12 antibiotics are shown in Table 4 and Additional file 4: Table S1.

**Table 4:** Antimicrobial resistance profiles of 145 *Vibrio parahaemolyticus* isolates from North China

Antimicrobial agent	*Vibrio parahaemolyticus* (*n* = 145)		
	NO. (%) of R^a^	NO. (%) of I^a^	NO. (%) of S^a^
Ampicillin (AMP)	72 (49.6)	40 (27.6)	33 (22.8)
Azitromycin (AZM)	0 (0.0)	27 (18.6)	118 (81.4)
Cefazolin (KZ)	63 (43.5)	75 (51.7)	7 (4.8)
Cephalothin (KF)	52 (35.9)	81 (55.9)	12 (8.2)
Chloramphenicol (C)	0 (0.0)	6 (4.1)	139 (95.9)
Ciprofloxacin (CIP)	3 (2.1)	9 (6.2)	133 (91.7)
Gentamicin (CN)	1 (0.7)	53 (36.5)	91 (62.8)
Kanamycin (K)	32 (22.1)	108 (74.5)	5 (3.4)
Nalidixic acid (NA)	3 (2.1)	1 (0.7)	141 (97.2)
Streptomycin (S)	125 (86.2)	17 (11.7)	3 (2.1)
Trimethoprim-sulfamethoxazole (SXT)	17 (11.7)	19 (13.1)	109 (75.2)
Tetracycline (TE)	2 (1.4)	22 (15.2)	121 (83.4)

^a^ *R*; resistant, *I*; intermediate resistance, *S*; susceptibility

## Discussion

In this study, we analyzed 260 aquatic product samples and detected *V. parahaemolyticus* contamination in 94 samples. Thus, the overall prevalence of *V. parahaemolyticus* in aquatic product samples was determined to be 36.2 %, which was in accordance with the results reported in a study from France [49] and in our previous study [18]. Notably, the prevalence of *V. parahaemolyticus* in summer (50.0 %) was higher than that in winter (22.7 %), and the levels of *V. parahaemolyticus* in the summer were significantly different from those in the winter. These results may be related to the differences in the average temperature of the two seasons. These observations were in agreement with the results of previous studies that showed a seasonal variation in the occurrence of this pathogen [50–52]. These results, which also confirm the conclusions of the WHO risk assessment for *V. parahaemolyticus* [53], can be useful for defining efficient monitoring programs in harvesting areas, based on temperature values for control of *V. parahaemolyticus*.

As the presence of *tdh-* and/or *trh*-positive *V. parahaemolyticus* strains in aquatic products represents a public health risk, their detection would be of paramount importance. In the present study, *tdh-* and/or *trh*-positive *V. parahaemolyticus* strains were not detected in any of the aquatic product samples. This finding is consistent with the findings of a previous study reported in India [54]. However, it is in contrast to the findings of other previous studies [55, 56]. The occurrence rate of these genes in pathogenic *V. Parahaemolyticus* isolates is high, as has been proven for clinical isolates. Isolates obtained from the environment and food contain much less *tdh* or *trh* than clinical isolates [55, 57]. However, it has also been shown recently that some clinical *V. parahaemolyticus* isolates do not possess *tdh* and *trh*. Even in the absence of these two hemolysins, *V. parahaemolyticus* remains pathogenic, indicating the existence of other virulence factors [29, 58].

As mentioned above, 13 O-serogroups and 71 K-serogroups have been identified in *V. parahaemolyticus*. The pathogenicity of *V. parahaemolyticus* strains varies and is associated with the serotype. Recently, a multiplex PCR-based O-antigen serotyping method was developed for detection and identification of *V. parahaemolyticus* [31]. This assay can effectively distinguish all *V. parahaemolyticus* O-serogroups, except O3 and O13. In the present study, nine O-serogroups were detected among the isolates. Our data indicated that serovar O2 was the predominant serotype among the strains isolated from the aquatic product samples, a finding that was in agreement with that of a study previously conducted by our group [18]. However, our findings were in disagreement with those of a previous study that identified the O3 serotype as the predominant serotype from shellfish from the eastern coast of China [17]. Previous study demonstrated that most *V. parahaemolyticus* outbreaks were caused by multiserovars of strains, mainly including O3:K6, O1:KUT, and O4:K68 [29, 30]. O3:K6, O1:Kut, O4:K8, and O2:K3 were also the dominant serovars of *V. parahaemolyticus*, that caused outbreaks in China [16, 59]. The relationship of serotype between the food poisoning isolates and the aquatic food isolates are of concern.

Recently, highly discriminatory molecular typing methods such as PFGE and ERIC-PCR have been developed for differentiation of pathogenic bacteria. ERIC-PCR is a relatively simple, cost-effective method. It is easier to perform than PFGE and is very useful for the analysis of large numbers of strains [38]. Using this approach in this study, the isolates were classified into 7 clusters, at 66 % similarity. This result is similar to those of other studies, confirming the genetic diversity within *V. parahaemolyticus* strains [60–62]. Some *V. parahaemolyticus* isolates were of the same types as the clinical strains and a reference strain, which may indicate that these strains are genetically related.

Susceptibility tests revealed that the isolates were resistant to some antibiotics. The highest resistance rate (86.2 %) was observed for streptomycin, followed by ampicillin (49.6 %), cefazolin (43.5 %), cephalothin (35.9 %), and kanamycin (22.1 %). Similarly, previous studies have shown that the occurrence of streptomycin- and ampicillin-resistance in *V. parahaemolyticus* isolates is common [63]. In the present study, a small number of isolates showed resistance to ciprofloxacin, gentamicin, nalidixic acid, and tetracycline, while none of the isolates demonstrated resistance to azithromycin, indicating that these antimicrobials were still highly effective against *V. parahaemolyticus* isolates. Based on our findings, these antibiotics could be prescribed by doctors for the treatments of *V. parahaemolyticus*. In our study, half of the isolates were resistant to more than three antibiotics. Increasingly, resistant strains are being reported [63, 64], which may be explained as follow: On one hand, along with the steady expansion of the Asian aquaculture industry, aquaculture farmers use many different antibiotics to prevent (prophylactic use) and treat (therapeutic use) pathogenic bacterial infections in aquatic produce [64, 65]. On the other hand, a wide range of antibiotics used in humans contaminate water, leading to resistance in pathogenic bacteria. In general, infection emergence of microbial resistance to multiple drugs is a serious clinical problem and can lead to an increase in fatality rates [65].

## Conclusions

This study showed that the levels of *V. parahaemolyticus* in retail aquatic products were relatively low and that none of the isolates possessed *tdh* and *trh*. Furthermore, serotype O2 was found to be the most prevalent; the isolates showed genetic diversity, as determined by ERIC-PCR typing, and the antimicrobial-resistance patterns showed that most of the isolates were resistant to streptomycin (86.2 %). The findings provided in this study may be useful in defining appropriate monitoring programs, understanding of the dissemination of antibiotic-resistant strains, and providing information for the assessment of exposure to this microorganism at the consumption level.

## Methods

### Bacterial strains

Two *V. parahaemolyticus* reference strains (ATCC 33847 and ATCC 17802) were obtained from the American Type Culture Collection (ATCC; Manassas, VA, USA). ATCC 33847 is *tdh*+, ATCC 17802 is *trh*+. Thirty-one clinical isolates were gifted by the Nanshan Shenzhen Center for Disease Control and Prevention (Shenzhen, China). All strains mentioned above were grown on Tryptone Soy Agar (TSA, Huankai Co. Ltd, Guangzhou, China) supplemented with 3 % (w/v) NaCl and incubated at 37 °C for 18 h.

### Sample collection of aquatic products

In total, 260 aquatic product samples, including 94 fish samples and 166 shrimp samples were collected in retail markets from 6 different cities in North China, belonging to 6 provinces, i.e., Harbin (*n* = 22 [winter], *n* = 22 [summer]), Lanzhou (*n* = 23 [winter], *n* = 22 [summer]), Xi’an (*n* = 22 [winter], *n* = 22 [summer]),Taiyuan (*n* = 22 [winter], *n* = 22 [summer]), Jinan (*n* = 21 [winter], *n* = 19 [summer]), and Beijing (*n* = 22 [winter], *n* = 21 [summer]). Samples were collected from November to December in 2012 and from July to August in 2013. In this region, the climate is cold from November to December (winter), and it is hot from July to August (summer). The samples were placed in sterile sealed plastic bags and transported to the laboratory in a cold box below 4 °C and were analyzed immediately.

### Most probable number (MPN) method for quantitative analysis

In this study, the MPN method was conducted accordance with the Bacteriological Analytical Manual standard and our previously study [18, 66]. Briefly, samples weighing 25 g were homogenized and combined with 225 mL of alkaline peptone water (APW) containing 3 % NaCl (Huankai, Guangzhou, China). Serial 10-fold dilutions were prepared up to a 1:10^3^ dilution, and 3 x 1 mL portions of each dilution were inoculated into 9 mL of APW with 3 % NaCl. Dilutions were incubating at 37 °C for 16–18 h. After incubation, the collected samples were streaked onto thiosulfate-citrate-bile salts-sucrose (TCBS) agar plates (Huankai, Guangzhou, China) with an inoculation loop and incubated at 37 °C for 18–24 h. Three to five (if have) presumptive *V. parahaemolyticus* colonies (green or blue green colonies, 2–3 mm in diameter) were selected from each plate, streaked onto Chromogenic Vibrio Medium (Huankai, Guangzhou, China) and incubated at 37 °C for 24 h. One (if have) mauve colony from each Chromogenic Vibrio Medium plate was selected for identification tests including halophilism tests, oxidase activity assessment, gram staining, the 3.5 % NaCl triple-sugar-iron (TSI) test, and API 20E diagnostic strips testings (BioMerieux Company, Marcy-l’Étoile, France) test. The total numbers of *V. parahaemolyticus* in samples were determined by converting the numbers of culture tubes positive for *V. parahaemolyticus* to MPN/g using an MPN table. The *V. parahaemolyticus* isolates were confirmed by amplifying *toxR,* as described previously [67].

### Detection of *tdh* and *trh* genes

Detection of the *V. parahaemolyticus tdh* and *trh* genes was performed by PCR, as described previously [68].

### Multiplex serotyping PCR

The serotypes of *V. parahaemolyticus* isolates were identified using the PCR-based O-antigen serotyping technique. The primer concentrations and amplification conditions used were as previously described [31].

### ERIC-PCR analysis

Genomic DNA was extracted from *V. parahaemolyticus* by using a commercial Universal DNA Extraction Kit (Sangon, Shanghai, China), according to the manufacturer’s instructions. Genomic DNA concentration was determined at 260 nm using a Nano Drop®ND-1000UVeVis Spectrophotometer (Thermo Fisher Scientific, Waltham, MA, USA). A pair of primers, ERIC 1R (5′-ATGTAAGCTCCTGGGGATTCAC-3′) and ERIC 2 (5′-AAGTAAGTGACTGGGGTGAGCG-3′) were used as previously reported [69]. ERIC-PCR typing was performed on the *V. parahaemolyticus* strains, using the protocol described previously with some modification [38]. More specifically, the reaction mixture (25 μL per reaction) consisted of 12.5 μL 2 × Long Taq Mix (Dongsheng Biotech, Guangzhou, China), 0.6 μmol/L of each primer, and 100 ng of template DNA. PCR was performed in a DNA thermocycler (Applied Biosystems, Foster City, CA, USA) by using the following cycling conditions: 1 cycle of denaturation at 95 °C for 5 min; followed by 35 cycles each consisting of 94 °C for 45 s, 52 °C for 1 min, and 72 °C for 3 min; and a final extension at 72 °C for 10 min. The PCR products were separated by electrophoresis in 2.0 % agarose gels, following which, they were subjected to GoldView staining (0.005 %, v/v) (SBS Genetech, Beijing, China) and photographed with a UV Imaging System (GE Healthcare, Waukesha, WI, USA). The images were captured in TIFF file format for further analysis.

### Antimicrobial susceptibility

The susceptibility of the *V. parahaemolyticus* isolates to antibiotics was examined by the disk-diffusion method, according to the guidelines of the Clinical and Laboratory Standards Institute [70]. Muller − Hinton agar and a panel of 12 antibiotics disks were selected for the resistance tests. These 12 antibiotic disks (Oxoid, Hampshire, UK) contained ampicillin (10 μg), azithromycin (15 μg), cefazolin (30 μg), cephalothin (30 μg), chloramphenicol (30 μg), ciprofloxacin (5 μg), gentamicin (10 μg), kanamycin (30 μg), nalidixic acid (30 μg), streptomycin (10 μg), trimethoprim − sulfamethoxazole (25 μg), or tetracycline (30 μg). The results were expressed as sensitive (S), intermediate (I), and resistant (R), following the methods of the CLSI. *Escherichia coli* ATCC 25922 and *V. parahaemolyticus* ATCC 17802 were used as quality control organisms.

### Statistical analysis

The size of each band in the ERIC patterns was determined and the data were coded as 0 (absence) or 1 (presence). Cluster analysis was performed with NTSYS-pc (Version 2.10), a numerical taxonomy and multivariate analysis software package [71], based on Dice’s similarity coefficient (SD), with a 1 % position tolerance and the unweighted-pair group method using arithmetic averages (UPGMA).

To facilitate statistical analyzes of quantitative data, half the detection limit (1.5 MPN/g) for the total *V. parahaemolyticus* levels in aquatic product samples was substituted when levels were below the limit of detection [52]. Significance of differences was determined by using SPSS 11.0 (IBM, USA) to perform an independent-samples *t*-test.

## Additional files

Additional file 1: Figure S1. ERIC-PCR1. M: DL2000; CK1: ATCC33847; CK2: ATCC17802. (PDF 153 kb) Additional file 2: Figure S2. ERIC-PCR2. M: DL5000; CK1: ATCC33847; CK2: ATCC17802. (PDF 142 kb) Additional file 3: Figure S3. ERIC-PCR3. M: DL5000; 1–31: SZ28-SZ58. (PDF 110 kb) Additional file 4: Table S1. Results of serotyping, antimicrobial resistance, and ERIC-typing of *Vibrio parahaemolyticus* isolates in this study. (DOC 221 kb)
