# Supplementary figures and images for: Prevalence, characterization, and antibiotic susceptibility of Vibrio parahaemolyticus isolated from retail aquatic products in North China
Source: BMC Microbiol. 2016 Mar 9;16:32. doi: 10.1186/s12866-016-0650-6 (PMC4784357; doi:10.1186/s12866-016-0650-6)

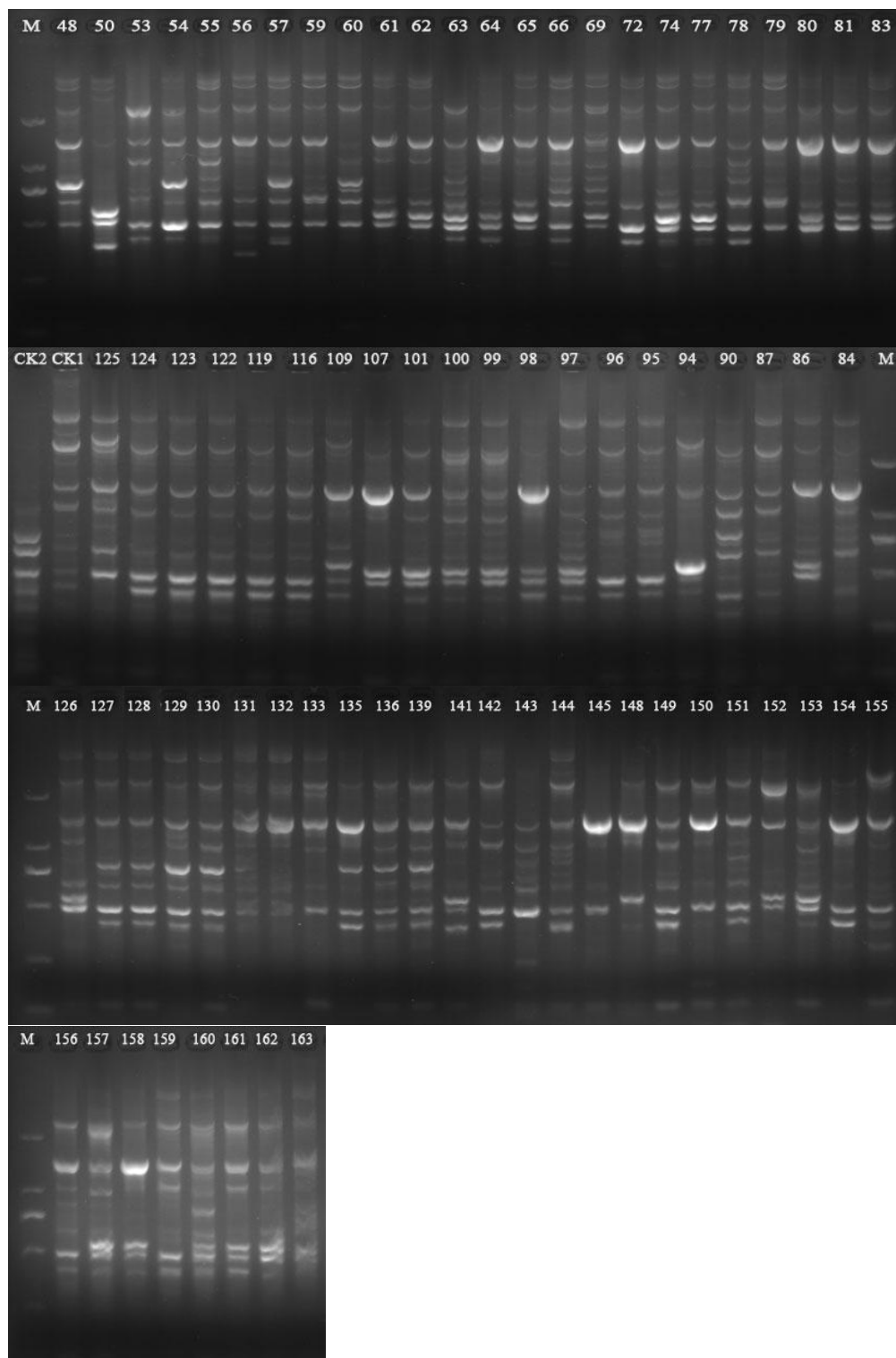

Supplement: Additional file 1: Figure S1. — ERIC-PCR1. M: DL2000; CK1: ATCC33847; CK2: ATCC17802. (PDF 153 kb) [file 12866_2016_650_MOESM1_ESM.pdf]

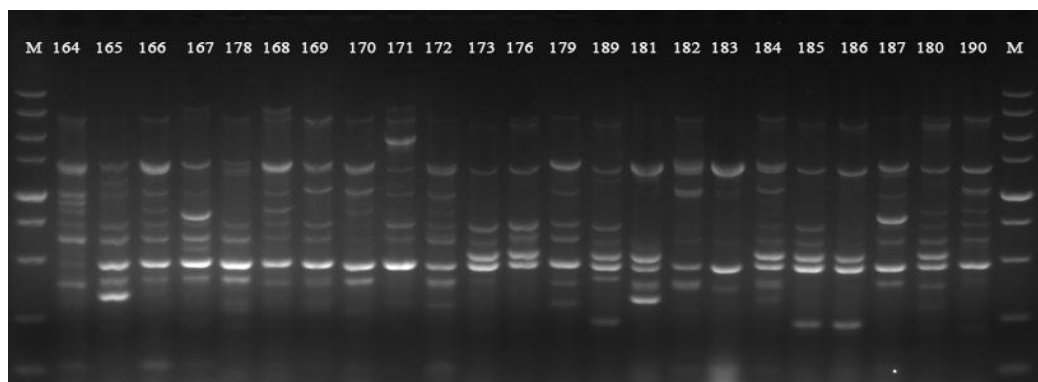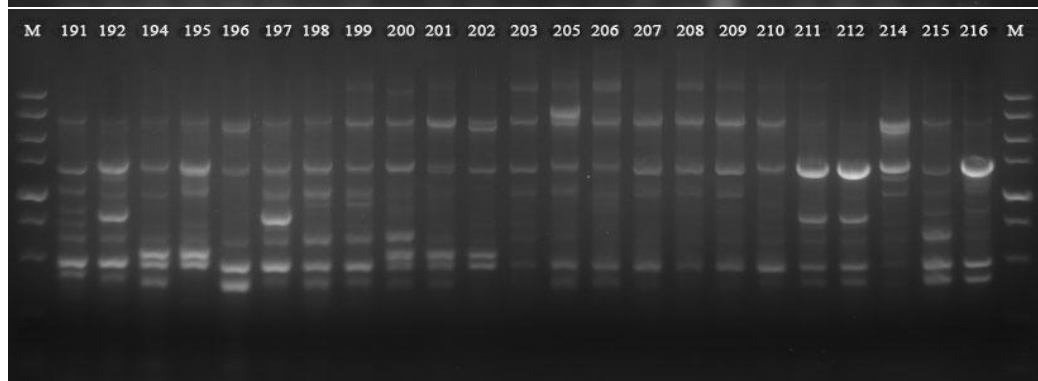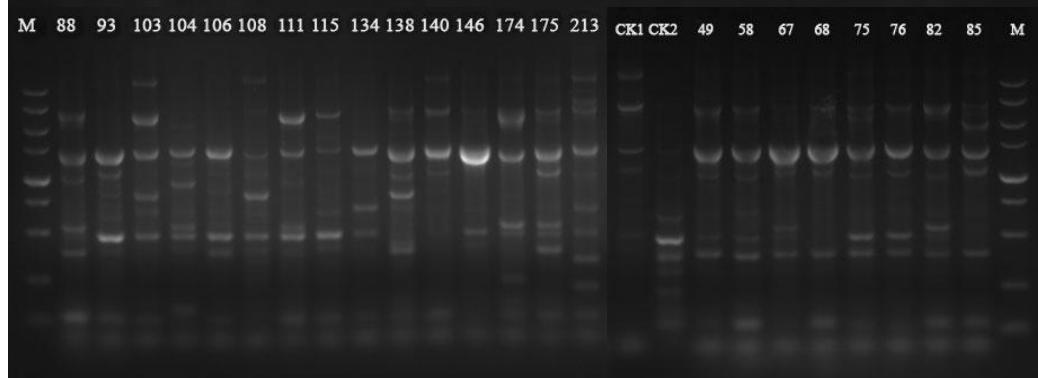

Supplement: Additional file 2: Figure S2. — ERIC-PCR2. M: DL5000; CK1: ATCC33847; CK2: ATCC17802. (PDF 142 kb) [file 12866_2016_650_MOESM2_ESM.pdf]

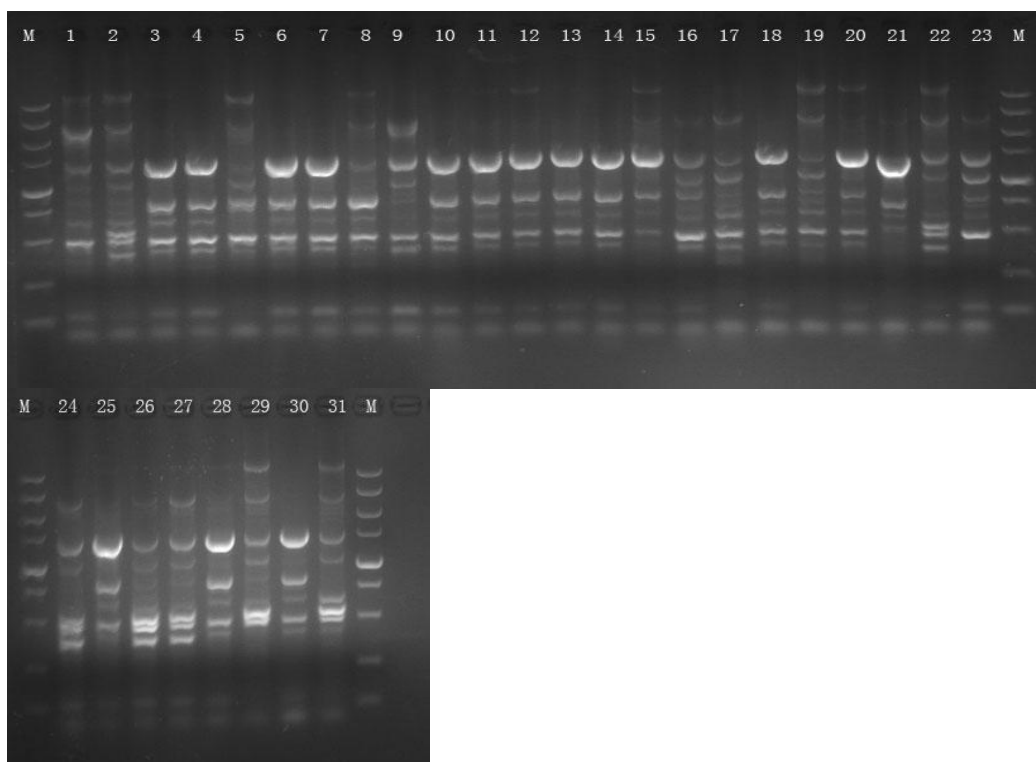

Supplement: Additional file 3: Figure S3. — ERIC-PCR3. M: DL5000; 1–31: SZ28-SZ58. (PDF 110 kb) [file 12866_2016_650_MOESM3_ESM.pdf]
