# Supplementary material for: Prevalence, characterization, and antibiotic susceptibility of Vibrio parahaemolyticus isolated from retail aquatic products in North China
Source: BMC Microbiol. 2016 Mar 9;16:32. doi: 10.1186/s12866-016-0650-6 (PMC4784357; doi:10.1186/s12866-016-0650-6)
Supplement: Additional file 4: Table S1. — Results of serotyping, antimicrobial resistance, and ERIC-typing of Vibrio parahaemolyticus isolates in this study. (DOC 221 kb) [file 12866_2016_650_MOESM4_ESM.doc]

**Supplementary Materials**

**Table S1. Results of serotyping, antimicrobial resistance, and ERIC-typing of *Vibrio parahaemolyticus* isolates in this study**

| Number | Source | Location | Serotyping^a^ | Resistance profile^b^ | ERIC |
| --- | --- | --- | --- | --- | --- |
| 48 | Shrimp | Lanzhou | O11 | S | A |
| 49 | Shrimp | Lanzhou | O11 | AMP-KZ-KF-K-S | D |
| 50 | Shrimp | Lanzhou | O8 | S | F |
| 53 | Shrimp | Lanzhou | uncertain | AMP-KZ | B |
| 54 | Shrimp | Harbin | O1 | AMP-S | A |
| 55 | Fish | Harbin | O11 | AMP-KZ-S | C |
| 56 | Shrimp | Harbin | O5 | S | A |
| 57 | Shrimp | Harbin | O1 | AMP-KZ-KF-S | A |
| 58 | Fish | Xi'an | O1 | AMP-KZ-KF-K-S-TE-SXT | A |
| 59 | Shrimp | Xi'an | uncertain | K-S | A |
| 60 | Shrimp | Xi'an | O5 | S | C |
| 61 | Shrimp | Xi'an | O2 | AMP-KZ-KF-K-S | C |
| 62 | Shrimp | Xi'an | O2 | AMP-KZ-K-S | C |
| 63 | Shrimp | Xi'an | O2 | AMP-S-SXT | B |
| 64 | Shrimp | Xi'an | O2 | S | E |
| 65 | Shrimp | Xi'an | O8 | AMP-S | C |
| 66 | Shrimp | Taiyuan | O2 | S | C |
| 67 | Shrimp | Taiyuan | O2 | KF-KZ-K-S-SXT | D |
| 68 | Shrimp | Taiyuan | O1 | AMP-KZ-KF-K-S-TE | D |
| 69 | Shrimp | Taiyuan | O1 | S | C |
| 72 | Shrimp | Taiyuan | O2 | AMP-S | A |
| 74 | Shrimp | Taiyuan | O2 | AMP-KZ-KF-K-S-SXT | A |
| 75 | Shrimp | Taiyuan | O1 | / | D |
| 76 | Shrimp | Taiyuan | O1 | AMP-KZ-KF-S | D |
| 77 | Shrimp | Taiyuan | O12 | AMP-KZ-K-S | A |
| 78 | Shrimp | Beijing | O2 | AMP-KZ-K-S-SXT | C |
| 79 | Shrimp | Beijing | O2 | AMP-KZ-S-SXT | A |
| 80 | Shrimp | Beijing | O2 | S-SXT | E |
| 81 | Shrimp | Beijing | O2 | KZ | E |
| 82 | Shrimp | Beijing | O2 | KZ-S-SXT | D |
| 83 | Shrimp | Beijing | O3 | S | E |
| 84 | Shrimp | Beijing | O1 | KF-S | C |
| 85 | Shrimp | Beijing | O12 | CIP-S-NA-SXT | D |
| Number | Source | Location | Serotyping | Resistance profile | ERIC |
| 86 | Shrimp | Beijing | O2 | AMP-K-S-SXT | B |
| 87 | Shrimp | Beijing | O1 | CIP-S-NA-SXT | C |
| 88 | Shrimp | Beijing | O2 | AMP-KZ-KF-S | E |
| 90 | Shrimp | Beijing | O1 | CIP-NA-SXT | C |
| 93 | Shrimp | Jinan | O1 | AMP-KF-S | B |
| 94 | Fish | Xi'an | O3 | KZ-S | C |
| 95 | Fish | Xi'an | O1 | AMP-KZ-KF-S | C |
| 96 | Fish | Xi'an | O1 | AMP-KF-S | C |
| 97 | Shrimp | Xi'an | O2 | KZ-S | B |
| 98 | Shrimp | Xi'an | O2 | S-SXT | E |
| 99 | Shrimp | Xi'an | O2 | S | C |
| 100 | Shrimp | Xi'an | O2 | KZ | C |
| 101 | Shrimp | Xi'an | O2 | S | E |
| 103 | Shrimp | Harbin | O5 | AMP-KZ | E |
| 104 | Fish | Harbin | O2 | AMP | E |
| 106 | Fish | Harbin | O12 | AMP-KZ-KF-S | B |
| 107 | Shrimp | Harbin | O2 | / | E |
| 108 | Fish | Harbin | O4 | AMP-KZ-KF-CN-K-S | A |
| 109 | Fish | Harbin | O3 | S | C |
| 111 | Shrimp | Harbin | O2 | AMP-KZ-KF-S | E |
| 115 | Shrimp | Harbin | O5 | S | E |
| 116 | Shrimp | Harbin | O3 | KZ-S | B |
| 119 | Shrimp | Harbin | O12 | AMP-KF | B |
| 122 | Shrimp | Harbin | O8 | AMP-KZ-KF-S | C |
| 123 | Shrimp | Harbin | uncertain | AMP-KZ | C |
| 124 | Shrimp | Harbin | O2 | KZ | C |
| 125 | Fish | Jinan | O8 | AMP-K-S | C |
| 126 | Fish | Jinan | O2 | S | C |
| 127 | Fish | Jinan | O8 | AMP-KZ | B |
| 128 | Fish | Jinan | O8 | / | B |
| 129 | Shrimp | Jinan | O8 | AMP-K-S | B |
| 130 | Shrimp | Jinan | O8 | S | B |
| 131 | Shrimp | Jinan | O2 | AMP-S | B |
| 132 | Fish | Jinan | O10 | AMP | C |
| 133 | Shrimp | Jinan | O12 | KZ-KF-S-SXT | C |
| 134 | Fish | Jinan | O4 | AMP-K-S | E |
| Number | Source | Location | Serotyping | Resistance profile | ERIC |
| 135 | Fish | Jinan | O8 | AMP-KZ-S | B |
| 136 | Shrimp | Jinan | O1 | AMP-KZ | B |
| 138 | Shrimp | Jinan | O1 | AMP-KZ-KF-K-S | B |
| 139 | Shrimp | Jinan | O1 | / | B |
| 140 | Shrimp | Jinan | O1 | KZ-KF-S | E |
| 141 | Shrimp | Jinan | O1 | S | E |
| 142 | Shrimp | Jinan | O1 | S | B |
| 143 | Shrimp | Jinan | O10 | S | B |
| 144 | Shrimp | Jinan | O1 | AMP-KZ-S | C |
| 145 | Shrimp | Jinan | O1 | / | B |
| 146 | Shrimp | Jinan | O4 | / | B |
| 148 | Shrimp | Jinan | O2 | AMP-S | E |
| 149 | Shrimp | Jinan | O3 | S | B |
| 150 | Shrimp | Beijing | O3 | AMP-S | B |
| 151 | Shrimp | Beijing | O2 | KZ-K-S | B |
| 152 | Shrimp | Beijing | O2 | KF-K-S | E |
| 153 | Fish | Beijing | O2 | AMP-KZ-S | B |
| 154 | Fish | Beijing | O11 | AMP-KZ-KF-S | B |
| 155 | Fish | Beijing | O3 | AMP-KF-S | B |
| 156 | Fish | Beijing | O11 | AMP-S | B |
| 157 | Shrimp | Beijing | O2 | AMP-KZ-K-S | E |
| 158 | Shrimp | Beijing | O2 | AMP-KZ-S | E |
| 159 | Shrimp | Beijing | uncertain | AMP-KZ-K-S | E |
| 160 | Shrimp | Beijing | O2 | AMP-KZ-K-S | B |
| 161 | Shrimp | Beijing | O2 | KF-S | E |
| 162 | Fish | Beijing | O2 | KZ-S | E |
| 163 | Fish | Beijing | uncertain | S | E |
| 164 | Fish | Beijing | uncertain | KF-S | B |
| 165 | Fish | Beijing | O5 | AMP-KF-K-S | B |
| 166 | Shrimp | Beijing | O11 | AMP-KZ-KF-S | B |
| 167 | Shrimp | Beijing | O11 | AMP-S | B |
| 168 | Shrimp | Beijing | uncertain | S | B |
| 169 | Shrimp | Beijing | O12 | AMP-S | B |
| 170 | Shrimp | Beijing | O3 | AMP-KZ-KF-S | B |
| 171 | Shrimp | Beijing | uncertain | S | B |
| 172 | Shrimp | Beijing | O1 | S | B |
| Number | Source | Location | Serotyping | Resistance profile | ERIC |
| 173 | Shrimp | Taiyuan | O2 | KF-K-S | B |
| 174 | Shrimp | Taiyuan | O2 | KZ-KF-K-S-SXT | E |
| 175 | Fish | Taiyuan | O2 | KZ-KF | E |
| 176 | Shrimp | Taiyuan | O2 | S | B |
| 178 | Shrimp | Taiyuan | uncertain | KZ-S | E |
| 179 | Shrimp | Taiyuan | O11 | KZ-KF-S | B |
| 180 | Shrimp | Taiyuan | O2 | KZ-S | B |
| 181 | Shrimp | Taiyuan | O2 | KF-S | G |
| 182 | Shrimp | Taiyuan | uncertain | K | B |
| 183 | Shrimp | Taiyuan | O1 | AMP-KZ-K-S | B |
| 184 | Shrimp | Taiyuan | O2 | AMP-KZ-KF-K-S | B |
| 185 | Shrimp | Taiyuan | O2 | KZ-KF-S-SXT | B |
| 186 | Shrimp | Taiyuan | O2 | S | B |
| 187 | Shrimp | Taiyuan | O8 | AMP-KZ-KF-S | B |
| 189 | Shrimp | Taiyuan | O2 | S-SXT | B |
| 190 | Shrimp | Lanzhou | O2 | AMP-KF-S | B |
| 191 | Fish | Lanzhou | O11 | S | B |
| 192 | Fish | Lanzhou | O8 | AMP-KZ-KF-S | B |
| 194 | Fish | Lanzhou | O2 | S | E |
| 195 | Fish | Lanzhou | O2 | KF-S | E |
| 196 | Fish | Lanzhou | uncertain | AMP-KZ-KF-K-S | E |
| 197 | Fish | Lanzhou | O1 | AMP-K-S | B |
| 198 | Fish | Lanzhou | O2 | KZ-K-S | E |
| 199 | Shrimp | Lanzhou | O2 | KF-S | E |
| 200 | Shrimp | Lanzhou | O2 | AMP-KZ-S | E |
| 201 | Shrimp | Lanzhou | O2 | AMP-KF-K-S | E |
| 202 | Shrimp | Lanzhou | O2 | KF-S | E |
| 203 | Shrimp | Lanzhou | O11 | KZ-S | E |
| 205 | Shrimp | Lanzhou | O1 | KF-S | E |
| 206 | Shrimp | Lanzhou | O11 | AMP-KZ-KF-S | E |
| 207 | Shrimp | Lanzhou | O11 | AMP-S | B |
| 208 | Shrimp | Lanzhou | O11 | AMP-K-S | E |
| 209 | Shrimp | Lanzhou | O11 | AMP-KF-S | E |
| 210 | Shrimp | Lanzhou | O4 | KF-S | E |
| 211 | Shrimp | Lanzhou | O4 | AMP-KF-S | B |
| 212 | Shrimp | Lanzhou | O11 | AMP-KZ-KF-S | B |
| Number | Source | Location | Serotyping | Resistance profile | ERIC |
| 213 | Shrimp | Lanzhou | O2 | S | E |
| 214 | Shrimp | Lanzhou | O5 | KF-S | B |
| 215 | Shrimp | Lanzhou | O11 | AMP-S | E |
| 216 | Shrimp | Lanzhou | O11 | AMP-KZ-KF-S | E |
| SZ28 | Clinical | Shenzhen | O1 | nd | E |
| SZ29 | Clinical | Shenzhen | O1 | nd | B |
| SZ30 | Clinical | Shenzhen | O3 | nd | B |
| SZ31 | Clinical | Shenzhen | O3 | nd | B |
| SZ32 | Clinical | Shenzhen | O3 | nd | B |
| SZ33 | Clinical | Shenzhen | O3 | nd | B |
| SZ34 | Clinical | Shenzhen | O3 | nd | B |
| SZ35 | Clinical | Shenzhen | uncertain | nd | A |
| SZ36 | Clinical | Shenzhen | O3 | nd | E |
| SZ37 | Clinical | Shenzhen | O1 | nd | B |
| SZ38 | Clinical | Shenzhen | O11 | nd | A |
| SZ39 | Clinical | Shenzhen | O1 | nd | A |
| SZ40 | Clinical | Shenzhen | O1 | nd | B |
| SZ41 | Clinical | Shenzhen | O3 | nd | B |
| SZ42 | Clinical | Shenzhen | O2 | nd | A |
| SZ43 | Clinical | Shenzhen | O4 | nd | B |
| SZ44 | Clinical | Shenzhen | O2 | nd | B |
| SZ45 | Clinical | Shenzhen | O4 | nd | B |
| SZ46 | Clinical | Shenzhen | O3 | nd | B |
| SZ47 | Clinical | Shenzhen | O3 | nd | A |
| SZ48 | Clinical | Shenzhen | O1 | nd | E |
| SZ49 | Clinical | Shenzhen | O3 | nd | E |
| SZ50 | Clinical | Shenzhen | O3 | nd | B |
| SZ51 | Clinical | Shenzhen | O2 | nd | E |
| SZ52 | Clinical | Shenzhen | O3 | nd | B |
| SZ53 | Clinical | Shenzhen | O11 | nd | B |
| SZ54 | Clinical | Shenzhen | O11 | nd | B |
| SZ55 | Clinical | Shenzhen | O3 | nd | B |
| SZ56 | Clinical | Shenzhen | O2 | nd | B |
| SZ57 | Clinical | Shenzhen | O3 | nd | B |
| SZ58 | Clinical | Shenzhen | O2 | nd | E |
| ATCC17802 | ATCC | USA | O1 | AMP-KZ-KF | A |

^a^O3 or O13

^b^For antimicrobial abbreviation, Azithromycin (AZM), Cefazolin (KZ), Cephalothin (KF), Chloramphenicol (C), Ciprofloxacin (CIP), Gentamicin (CN), Kanamycin (K), Nalidixic acid (NA), Streptomycin (S), Trimethoprim-sulfamethoxazole (SXT), Tetracycline (TE); /, no resistance; nd: not determined
